# Health and human rights of women imprisoned in Zambia

**DOI:** 10.1186/1472-698X-11-8

**Published:** 2011-06-22

**Authors:** Katherine W Todrys, Joseph J Amon

**Affiliations:** 1 Human Rights Watch, New York, USA

## Abstract

**Background:**

The healthcare needs and general experience of women in detention in sub-Saharan Africa are rarely studied and poorly understood.

**Methods:**

A mixed-methods study was conducted including in-depth interviews with 38 adult female prisoners and 21 prison officers in four Zambian prisons to assess the health and human rights concerns of female detainees. Key informant interviews with 46 officials from government and non-governmental organizations and a legal and policy review were also conducted.

**Results:**

Despite special protection under international and regional law, incarcerated women's health needs–including prenatal care, prevention of mother-to-child transmission of HIV, and nutritional support during pregnancy and breastfeeding–are not being adequately met in Zambian prisons. Women are underserved by general healthcare programs including those offering tuberculosis and HIV testing, and reported physical and sexual abuse conducted by police and prison officers that could amount to torture under international law.

**Conclusions:**

There is an urgent need for women's healthcare services to be expanded, and for general prison health campaigns, including HIV and tuberculosis testing and treatment, to ensure the inclusion of female inmates. Abuses against women in Zambian police and prison custody, which violate their rights and compromise their health, must be halted immediately.

## Background

In African countries, female prisoners comprise between one percent (in Burkina Faso) and 6.3 percent (in Mozambique) of the total convicted population [1]. Like their male counterparts, women in African prisons frequently face overcrowded and unsanitary conditions conducive to poor health and the spread of infectious disease [1]. Yet women are also confronted with unique challenges–related to menstruation; pregnancy and childbirth; care for children both inside and outside of prison; and violence and abuse (including sexual abuse) by prison officers or male prisoners with whom they are sometimes held [1]. Women prisoners often have experienced violence and sexual abuse prior to incarceration, and may suffer from post-traumatic stress disorders while detained [2]. Despite recognition of these challenges, there has been little research on this population to date.

In Zambia, female inmates comprise approximately 1.5 percent of the total prison population, with an estimated 250 female prisoners incarcerated nationwide in March 2011 [3,4]. While one of Zambia's 86 prisons is dedicated exclusively to female inmates, and housed 66 women in March 2011 [4], women live in separate sections of prisons located throughout the country.

In addition to the international human rights law protections afforded to all prisoners and all women, female detainees benefit from special legal protections. African regional law, for example, provides that women in detention should be held in an environment "suitable to their condition" and ensures their right to be treated with dignity [5]. The Southern African Development Community Protocol on Gender and Development, which Zambia has signed, commits states by 2015 to "ensure the provision of hygiene and sanitary facilities and nutritional needs of women, including women in prison" [6].

This study examines women prisoners' experiences of imprisonment in Zambia, with particular emphasis on access to HIV and tuberculosis (TB) prevention, testing, and treatment, women's unique healthcare needs, and treatment in police and prison custody. The main objective of the investigation was to document and respond to specific human rights issues, monitor human rights conditions, and assess human rights protections in Zambian prisons. Analysis of male inmates' conditions of detention and the criminal justice system attributes leading to their extended detention in Zambian prisons has been published elsewhere [7,8].

## Methods

In July 2009, the Prisons Care and Counselling Association (PRISCCA) of Zambia sought approval from the Zambian Ministry of Home Affairs and Ministry of Foreign Affairs for a mixed-method study of health conditions in Zambian prisons. In September 2009 both Ministries granted permission. Between September 2009 and February 2010, researchers from PRISCCA, the AIDS and Rights Alliance of Southern Africa (ARASA) and Human Rights Watch conducted in-depth interviews with female prisoners in four Zambian prisons: Lusaka Central Prison (Lusaka province), Kamfinsa State Prison (Copperbelt province), Mumbwa Prison (Central province), and Choma State Prison (Southern province).

In each prison visited, researchers requested from the officer in charge a private location to conduct interviews. Officers identified prisoners who were then provided by researchers with a verbal explanation of the research (in English or French, and translated into Bemba, Nyanja, or Tonga if necessary), asked if they were willing to participate, and offered anonymity. Individuals were assured that they could decline to participate, end the interview at any time, or decline to answer any specific questions without negative consequence. The names of all prisoners who participated in this study have been changed to protect their anonymity and security. Responses were recorded in written notes and all interviews were conducted outside of the hearing of prison officers and other prisoners, in a private setting. The Zambia Prisons Service did not permit the use of recording devices.

Interviews took approximately 45 minutes and were conducted in English or French by female researchers from one of two organizations (Human Rights Watch or ARASA) or in Bemba, Nyanja, and Tonga, with translation to English provided by members of PRISCCA. Interviewers used a brief verbal questionnaire to gather information on the prisoner's incarceration history, medical care, and experience of HIV/AIDS and TB testing and treatment. Researchers then probed responses and asked further questions regarding prison conditions, discipline, and HIV/TB risk behavior in open-ended, in-depth interviews.

Qualitative prisoner data were hand-coded by the authors, who conducted a content analysis to identify key themes corresponding to the interview guide, as well as emergent topics. In the first analysis of the data, an initial set of codes was generated to capture key constructs. Subsequent analyses were undertaken to examine the consistency of reports across themes and examine negative evidence [9].

At each facility visited, researchers also requested interviews with the officer in charge, deputy officer in charge, medical officer and female officer in charge; additional officers were invited to participate if sufficient time allowed. Prison officers were provided with an explanation of the purpose of the study and how the information obtained would be used; they were given the opportunity to decline the interview or to end the interview at any time. Prison officer interviews focused on HIV and tuberculosis testing and treatment availability in the prison, healthcare delivery, deaths in custody, prison administration, prisoner discipline and treatment, and prison officers' working conditions.

Interviews with key informants from government and national and international non-governmental organizations (NGOs) were also conducted, prior to and following prison-based interviews, to identify salient issues and probe specific findings raised in the research.

In addition, national legislation and policy governing the administration of the prison and criminal justice systems were reviewed. Researchers reviewed all existing Zambian legislation, extracting relevant portions related to prison administration, criminal procedure, criminal law, and immigration law. All available national policies related to HIV/AIDS, health, and prison administration were also examined.

Human Rights Watch does not generally identify its work as "research", defined as seeking to develop "generalizable knowledge" [10]. Rather, its investigations aim to document and respond to specific human rights issues, monitor human rights conditions, and assess human rights protections in Zambian prisons. Each of these purposes is consistent with what has been defined as "public health non-research" [11] or practice [10]. However, because public health non-research and practice also raise ethical and human participant protection issues, all investigations conducted by Human Rights Watch are subject to rigorous internal review, and external ethics and subject-area experts are consulted when investigations involve particularly difficult settings, populations, or issues. The present study's methods, and human participant protections associated with the research, were reviewed by PRISCCA, ARASA, and Human Rights Watch prior to undertaking this study, and all interviewers were trained in human participant protection and information security. Following the interviews, and after initial reports of the study had been released, PRISCCA and Human Rights Watch continued to monitor to ensure no adverse consequences to subjects from participation.

## Results

Thirty-eight adult female prisoners from four Zambian prisons were interviewed, including 20 convicted, 14 pre-trial, and four immigration detainees. Their ages ranged from 22 to 77 years, with an average age of 37 years. Thirty-two of the 38 women (84 percent) were of Zambian nationality. Female prisoners interviewed reported having been detained at the facility at which they were interviewed for an average of nine months (with a range from zero to 44 months). Twenty-one (55 percent) of the women had reached secondary-level education or higher, while six (16 percent) had received no education and 11 (29 percent) had only a primary-level education. In addition, 22 prison officers and 18 Zambian government officials from relevant ministries were approached for interviews; one prison officer declined. Twenty-eight representatives from local and international NGOs and donor governments and agencies were also interviewed.

### General Conditions

Women in Zambian prisons live in conditions of severe overcrowding. Zambian prisons are over 300 percent of capacity, and female inmates reported sleeping four to a mattress, packed together in unventilated cells with young children and the sick [12]. As one female inmate reported: "Our cells are normally stuffed. There is no ventilation, no windows. The sick and healthy are mixed up. There are those with diarrhea. We are breathing the same air" [13].

Both prisoners and prison officials reported that the food provided by the government to prisoners is insufficient and nutritionally inadequate. Prisoners rely on their relatives to supplement the meager food rations or trade work for food. As one female inmate noted, "Some people have no relatives–if you have no food, you are nobody in this place. You can trade a cup of sugar for work" [14].

The Zambia Prisons Service does not provide inmates with basic necessities including soap, toothpaste, or sanitary pads. As one inmate noted, "If others don't bring them for us, we have nothing. There are lots of people with no relatives here. They have nothing" [14]. Female prisoners reported that sanitation and hygiene are poor, and water frequently unclean. "It tastes foul, but we drink it", stated one female HIV-positive inmate [15].

In April 2010 only 14 health personnel served 16,666 prisoners, and of Zambia's 86 prisons, only 15 had any health clinic or sick bay [3]. For those at prisons without a clinic–and for those with more serious medical conditions at those with a clinic–access to care is controlled by medically unqualified and untrained prison officers. According to prisoners and prison officers, a lack of adequate prison staff for the transfer of sick prisoners, inadequate vehicles for transportation and fuel, and security fears keep inmates from accessing medical care outside of prisons, in some cases for weeks after they fall ill. As one inmate reported:

"There are delays in getting to the clinic. It depends on the officials, if they want to take you there or not. Sometimes you can go as long as a month waiting to go to the clinic....They don't open the door in the cell at night for anything. There are no windows, no air. Someone who was 28 years old died at night in my cell and they didn't open the door until the morning" [16].

### Pregnancy and women with children

International standards dictate that for women in detention, there shall be "special accommodation for all necessary prenatal and postnatal care and treatment" [17]. Zambia Prisons Service policy requires that "Women inmates, including those who are HIV infected, should receive...[p]rovision of antenatal care services as offered to all women in the general population" [18]. But although prenatal care is widely available in the Zambian general population [19], incarcerated pregnant women interviewed described inadequate, and in some cases non-existent, prenatal care. One pregnant woman reported: "I had no initial exam when I came to the facility, even though I am pregnant. There is no special treatment for pregnant women, I take whatever I can" [20]. Another female inmate, who reported she was six months pregnant, said:

"I have not been to the clinic yet, no antenatal care. I went to the clinic once but was told the nurses were not working. Since then I have not asked. I do not feel well, lots of ups and downs" [21].

For some prisoners, prenatal care existed but did not meet international standards. The World Health Organization (WHO) protocol for prevention of mother-to-child transmission (PMTCT) of HIV notes that "[a]ll HIV-infected pregnant women who are not in need of ART [antiretroviral therapy for HIV treatment] for their own health require an effective ARV prophylaxis strategy to prevent HIV transmission to the infant. ARV prophylaxis should be started from as early as 14 weeks gestation" [22]. However, one female prisoner, who was eight months pregnant, reported:

"I had VCT [voluntary counseling and testing for HIV]–they tested my blood again and told me I was HIV-positive. They told me my CD4 court was too high for ART. I wasn't given any HIV drugs to prevent transmission, only folic acid and vitamins" [23].

Indeed, there is no PMTCT program under the prison medical directorate, though PMTCT programs have been scaled up in recent years in the general population. The estimated percentage of women in Zambia's general population living with HIV who received ART for prevention of mother-to-child transmission increased between 2004 and 2007 from 18 to 47 percent [24].

Inadequate nutrition is a serious problem for pregnant women and women with children in prison. Prisoners across facilities reported that meals consisted of approximately 400 to 450 grams of maize meal per day (400 grams of maize meal is equivalent to roughly 1,400 calories [25]), in addition to small quantities of beans and/or kapenta (tiny fish commonly eaten in Zambia). Normal-weight pregnant women require between 1,900 and 2,500 calories per day during the last six months of pregnancy for healthy weight gain [26]. Yet there is no special diet for pregnant women or for women who are nursing.

Despite international standards calling for special provisions for children incarcerated with their parents [27] and Zambian law, which states that, "the infant child [up to age four years] of a woman prisoner may be received into the prison with its mother and may be supplied with clothing and necessaries at public expense" [18,28,29], the Prisons Service allocates no food to children who live with their mothers in prison facilities. In situations where women are unable to breastfeed, the prison does not offer infant formula. As the officer in charge at one prison reported: "I get no budget for the children's food, they must eat their mothers' food. They are hungry a lot" [30]. As the incarcerated mother of a nine-month-old boy said:

"My child is not considered for food–I give my share to the baby, beans and kapenta–we each eat once a day. I am not given any extra food, and no special diet for the child. I simply make some porridge for him out of my nshima. The baby has started losing weight and has resorted to breast milk because the maize meal is not appetizing" [31].

Another female inmate said: "I am worried about the children who are here. There was a baby who died. They don't pay any particular attention to the children. They are mixed in with everyone, they don't have their own cell or better food" [21].

### HIV and TB testing

HIV and TB are major health threats for the entire prison population–when last measured in 1999, HIV prevalence was 27 percent for the Zambian prison population, and 33 percent among female inmates [32]. Whereas TB prevalence was estimated to be 0.3 percent in the Zambian general population in 2009 [33], a 2000-2001 study in 13 Zambian prisons estimated the prevalence of pulmonary TB to be between 15 and 20 percent [34]. HIV testing and treatment are offered at six prisons nationwide with the assistance of an NGO, and as of March 2011, prison-based TB screening and treatment were offered only at three prisons nationwide as part of a pilot program. For both diseases, researchers found that female inmates were less likely to have been tested than their male counterparts [8].

Female prisoners face potential breaches of consent with HIV testing. The National HIV/AIDS/STI/TB Policy requires that women considering having a child be encouraged to seek counseling and testing and ensures that every pregnant woman has access to HIV/STI screening and treatment. It does not require mandatory prenatal testing [35]; Zambia Prisons Service policy prohibits compulsory HIV testing [18]. However, interviews with prison officers suggested that this prohibition was either not understood or not respected for female inmates. One prison officer said: "For those who are pregnant, they are tested for HIV....Whether you like it or not you are tested to prevent transmission to the baby" [36].

### Abuse of female inmates in police and prison custody

Female inmates, particularly women previously held in police custody, reported physical and sexual abuse indicative of a widespread and systematic pattern of brutality. Prisoners repeatedly reported that they were beaten in police custody in order to try to coerce a confession, often leading to serious injuries. One female inmate who had previously been held in police custody, said:

"When I was in police custody, they beat me, a torture I have never experienced in my lifetime. They beat me, undressed me, whipped me. They put handcuffs on me so hard that the blood couldn't flow. They turned me upside down and hung me upside down, with a steel cord between my legs. They swung me and beat me. They saw I was crying and screaming and put a cloth in my mouth to suffocate me. I fainted–I couldn't handle the pain. They were abusing me with their language, calling me a prostitute. They put me somewhere where I couldn't talk to anyone. They were trying to get me to say something–I don't know. They were just torturing me for four days, beating me. After, there was lots of blood where I was beaten. My hands were green and swelling.

They hit me on my ears and face with a metal band. There were scratches on my face. They said, 'you have to give us information about who had killed the person'. They tried to find out who had killed the person–I didn't know. The police are supposed to investigate a case, not to torture.

After, they were scared to take me to a doctor because I still had injuries. They only took me after one month, when the swelling was down. When I went to the doctor, the police [officer] followed me into the doctor's room and listened to me. The police told the doctor that I was lying. 'Just a simple torture that she was given, not much,' he said" [13].

Several female detainees reported that police officers tried to coerce them into sex in exchange for their release. One female prisoner who had been detained in police custody reported:

"They arrested and they beat me, asking questions. They beat me up when I said I didn't know anything. They said, 'we want you to say this, then we will let you go.' They didn't sexually abuse me, but they asked me to have sex with them. They said they would release me if I did, and I said no" [37].

Female inmates also reported particularly brutal forms of punishment that they were subjected to once incarcerated in prisons. Women in prison were subjected to beatings by their fellow inmates at the instigation of the officers:

"When I arrived here, the officers shouted to the inmates to say, 'the woman coming is a witch, a murderer–deal with her!' Shouting 'you witch, you murderer,' they rose up, hitting me. The officers just watched. My injuries were largely bruises and swellings on my arms where I was beaten....I felt humiliated and dehumanized to the extent that I almost committed suicide. I couldn't bear it" [38].

Reported another female inmate:

"Truthfully, each officer has her own problems. Some are harsh, some don't accommodate us. To tell the truth, we were told to say that there are no problems, but each has their own problems. They beat and shout at us, so we can't share a problem–they are not approachable....They degrade us, shout, call us names, make reference to the fact that we are criminals" [31].

In addition to beatings, at one prison, female prisoners reported sexual humiliation as punishment. Prisoners reported being stripped naked, smeared with mud, and placed in the direct sunlight of the prison central courtyard to be viewed by all female prisoners for an entire day as punishment. One female inmate described this punishment as "aimed at humiliating or insulting our personality" and asked: "How can they make me strip naked before younger women who could be my daughter, without taking to consideration how I would feel as a woman, as a mother?" [38]

Further forms of sexual humiliation and verbal abuse exist for female inmates. One inmate reported that as punishment, the officers may put the inmate into the center of a circle of the other prisoners at bath time, where each "showers insults at her, calling her the names of private parts" [15].

Female prisoners also reported feeling abused by routine strip searching by officers. Inmates reported that they were strip searched, both when returning from court, and at regular intervals in the cells themselves. Multiple inmates reported the shame involved. "I feel grieved about it", one said, "I even pray to God that I can just die. The pain and shame is too tough to bear" [38]. In one instance, researchers received a report from a prisoner that a body cavity search for all female inmates was carried out with a single pair of gloves [13].

## Discussion

This study is the first independent research conducted on issues related to the health and human rights of female detainees in Zambian prisons. Our findings indicate that both the healthcare available to and the abuse endured by women in detention in Zambia violate international human rights law. Women in Zambian prisons are not provided with healthcare services to address their specific health needs, and are underserved by the HIV and TB testing which does exist.

While there has been relatively little research conducted to date addressing the distinct healthcare needs faced by women in detention worldwide [2,39,40], and even less in Africa, news accounts suggest major problems, including: poor conditions and no medical care for women detained in a Sudanese prison [41]; physical abuse against women in a Mauritius prison [42]; and rape and sexual violence against female detainees by male inmates in the Democratic Republic of the Congo [43].

Understanding the human rights abuses impacting female prisoners' health in incarceration facilities is key to devising appropriate, effective services for this population, and may become increasingly important in coming years: Female prison populations have increased in both absolute numbers and in proportion to the male population in some countries recently [44].

Under international human rights law, people in detention retain their human rights and fundamental freedoms–aside from such restrictions on their rights as are required by the fact of incarceration [17,45,46] and except as necessary for the justifiable segregation or the maintenance of discipline [17]. States have an obligation to ensure medical care for prisoners at least equivalent to that available to the general population [47-49]. The current lack of access to health care–evidenced by the lack of facilities in Zambian prisons and the evaluation of medical needs by untrained and unqualified guards–falls far below the equivalent standard of available treatment in the community. Further, the Convention on the Elimination of All Forms of Discrimination against Women obligates states to ensure to all women–in and outside of prison–appropriate services for pregnancy, including adequate nutrition [50].

Respecting female detainees' human rights is also essential to preventing abuse and ensuring the health of female detainees. The most fundamental protection for detainees is the absolute prohibition on torture, inhuman or degrading treatment or punishment. In addition to being a well-established norm of international law by which Zambia is bound, the prohibition is also reflected in the Zambian Constitution and in several of the human rights treaties to which Zambia is a party [47,48,51,52]. Yet the physical abuse suffered by Zambian female detainees in police and prison custody violate national and international law prohibitions on torture or cruel, inhuman or degrading treatment or punishment, as well as having negative consequences for their health.

There were several limitations to our research. Women prisoners in only four of 86 prisons nationwide were interviewed, and the recruitment of prisoners required cooperation of prison officers. Researchers did not have access to the one female prison in Zambia, and so were restricted to interviewing female detainees held in primarily male prisons. Because the prisons where interviews took place were participating in an ongoing HIV prevention program run by a non-governmental organization (PRISCCA), and subject to visits by NGO staff, conditions may have been better than in prisons not participating in the program. Similarly, the selection of prisoners by prison officers likely biased the sample to healthy prisoners not currently in punishment cells, who were possibly more likely to portray prison staff and conditions in a positive light.

However, using mixed-method approaches and triangulating information from prisoners with in-depth interviews with prison officers and NGO and government representatives strengthened our confidence in our main findings. Even if our results suggest more positive conditions than those experienced by a more representative sample of female Zambian prisoners, the findings identify serious human rights abuses and failures to provide healthcare that compel further investigation, monitoring and response by the Zambian government.

## Conclusions

Although women detainees are entitled to protection under international human rights law and enjoy specific protections under regional human rights standards, in Zambia, incarcerated women's specific health needs are not being met, and they are underserved by general health services. There is an urgent need for healthcare services targeted to female inmates, including prenatal care, to be expanded, and for general prison health campaigns including HIV and TB prevention, testing and treatment to include women prisoners. Abuses against women both in police and prison custody which violate their rights and compromise their health must be eliminated.

## Competing interests

The authors declare that they have no competing interests.

## Authors' contributions

KWT and JJA conceived the study. KWT led the field research and KWT and JJA drafted the manuscript. Both authors read and approved the final manuscript.

## Pre-publication history

The pre-publication history for this paper can be accessed here:

http://www.biomedcentral.com/1472-698X/11/8/prepub
